# Shannon Entropy and Beyond: An Information-Theoretic Framework for Randomness Pre-Screening

**DOI:** 10.3390/e28060695

**Published:** 2026-06-16

**Authors:** Alexandru Dinu

**Affiliations:** Faculty of Electronics, Telecommunications and Information Technology, National University of Science and Technology POLITEHNICA Bucharest, 061071 Bucharest, Romania; alexandru.dinu2408@upb.ro

**Keywords:** Shannon entropy, Rényi entropy, permutation entropy, sample entropy, PRNG validation, lottery data, machine learning

## Abstract

Shannon entropy is the most common measure that one could use to check if a data source has random behaviour or not. A value close to the maximum is usually considered as evidence that the source is “random enough”. The present paper shows that this criterion alone is not enough. A deterministic logistic map driven at r=3.9999 reaches 94.97% of the Shannon maximum, yet it is fully predictable once we look at the built-in patterns: its permutation entropy drops to 77.01% of the maximum and its sample entropy falls to 0.67, against 2.33 for a high-quality pseudo-random generator (PRNG). Building on this observation, we combine four entropy measures—Shannon, Rényi, permutation, and sample—into a single diagnostic profile of the analyzed source. In order to validate our approach with practical, real life data, we test it on 2538 official draws of the Romanian Loto 6/49 lottery, recorded between August 1993 and April 2026. The lottery historical data set is very close to a high-quality PRNG (pseudo-random number generator) from the point of view of all four measures. We also observe that the entropy deficit of both the lottery and the PRNG decays as a power law with exponent α≈−0.96; in contrast, the logistic map sits at α≈−0.07. A Random Forest classifier trained only on the entropy profile reaches 78% accuracy on the analyzed four-way classification task (lottery, PRNG, logistic map, biased distribution), but scores 55.7% on the binary lottery-versus-PRNG task, consistent with chance. The method introduced in this study is domain-independent and applies directly to RNG certification, cryptographic key auditing, and any setting where structured pseudo-randomness has to be ruled out.

## 1. Introduction

Deciding if a data source behaves in a random fashion is an important recurring problem in multiple areas of research such as cryptography, statistics, and physics. Shannon entropy [1] is the go-to tool that most scholars make use of in such cases. A source is typically declared “random enough” when its empirical Shannon entropy is very close to the theoretical maximum $H_{\max}=\log_{2}M$, where *M* is the alphabet size.

This approach does not work for a simple case presented next. Consider the logistic map $x_{t+1}=rx_{t}\left(1-x_{t}\right)$ at r=3.9999, discretised to an alphabet of size M=49. The sequence produced by the chaotic system is entirely deterministic, yet its Shannon entropy reaches 94.97% of $H_{\max}$. Most of the entropy threshold-based tests would accept this as a random source. Nevertheless, permutation entropy [2] and sample entropy [3] behave differently: they analyze how symbols follow each other, not just how often each symbol appears. On these measures, the same sequence based on the logistic map drops to 77.01% of the maximum permutation entropy and a sample entropy of 0.67, compared to a reference value of 2.33 for a high-quality PRNG. The deterministic structure from the chaotic system is hiding in the sequence, and Shannon entropy is unable to detect it alone.

Our previous work [4] showed that machine learning models cannot accurately predict Romanian Loto 6/49 draws, and in any case is not better than making random choices. However, the draw sequence passes the usual uniformity and independence tests. A natural follow-up question is if this conclusion is valid under stricter analysis. If Shannon entropy can be misled by a chaotic map, perhaps the lottery hides structure too, just not of the kind Shannon entropy detects. This was the motivating question we started our investigation from.

The paper is organised around three main points:

1. We combine four entropy measures—Shannon, Rényi, permutation, and sample entropy—into a single profile, and show that both the lottery data and a PRNG score almost the same across all four criteria mentioned above.
2. We observe that the entropy deficit—the fractional gap between the observed and the maximum entropy—of the lottery and PRNG decays as a power law in the number of observations, with exponent α≈−0.96. The logistic map is at α≈−0.07. This gap is large enough to be used as a practical criterion to check pseudo-randomness.
3. We train a Random Forest classifier on eight entropy-based features computed over sliding windows of size 300 symbols. This is able to separate the logistic map and a biased distribution from the rest with very good accuracy, but is not able to distinguish between the lottery and the PRNG.

Section 2 covers related work and introduces the four entropy measures and illustrates each one with a small numerical example from the lottery data. Section 3 describes the dataset and the three reference sources we use for comparison. Section 4 introduces the combined entropy profile and the Rényi spectrum. Section 5 analyses how the entropy deficit evolves depending on sample size. Section 6 focuses on permutation entropy as the most discriminating of the four measures. Section 7 describes the machine learning experiment. Section 8 discusses implications and limitations. Section 9 concludes the paper.

## 2. Background and Related Work

### 2.1. Randomness Testing

The NIST Statistical Test Suite [5] is the standard battery for evaluating PRNGs for cryptographic applications. The set contains 15 tests covering frequency, runs, spectral, and complexity evaluations. The Diehard battery [6] was introduced before NIST, and it is still used as a benchmark. Both setups produce pass/fail results, with no continuous quantitative measure that would describe how close or how far a source is to ideal randomness.

Approaches based on entropy evaluation have been proposed as complements to the above-mentioned test batteries. Paninski [7] showed that the plug-in Shannon estimator is negatively biased for small sample size and proposed several corrections. The Miller–Madow correction [8] and the Grassberger correction [9] address the same issue from different perspectives. These corrections are important for the convergence analysis performed in Section 5.

Bandt and Pompe [2] introduced permutation entropy as a measure to evaluate complexity for time series. Zanin et al. [10] investigated the use of this parameter in biomedical signals and econophysics. Cao et al. [11] stated that it is able to detect dynamical changes more reliably than Lyapunov exponents when there is noise present. Riedl et al. [12] compared the permutation entropy with approximate entropy and sample entropy and concluded that permutation entropy manages noisy scenarios better but is less sensitive to amplitude.

#### 2.1.1. Entropy and Chaos

The connection between entropy and chaotic dynamics is studied and well understood. The Kolmogorov–Sinai entropy [13] is the theoretical upper limit for the entropy rate of a dynamical system. Based on Pesin’s theorem, it is equal to the sum of the positive Lyapunov exponents. For the logistic map with r=3.9999 the Kolmogorov–Sinai entropy is positive, so the system is genuinely chaotic. However, the distribution of the values that the map produces is close to the uniform distribution, which is why Shannon entropy alone does not detect the determinism.

Our own previous work on Lorenz and Rössler systems [4,14,15] found that chaotic systems can pass first-order statistical tests while still carrying structure that can be exploited. The present paper extends that investigation and transforms it into a systematic framework for pseudo-randomness validation.

#### 2.1.2. Lottery Data Set

There have been several studies in the past which have applied statistical tools to lottery data. Wang et al. [16] looked at number preferences among players and they could not identify an exploitable structure in the lottery draws. Farrell et al. [17] investigated how demand patterns behave based on the UK National Lottery system. Our own previous work [4] applied a joint treatment of statistical testing and machine learning techniques on Romanian Loto 6/49 data. The present paper advances new ideas and moves beyond a single-measure Shannon analysis to a four-measure profile. In addition, it adds a convergence-based check.

### 2.2. Four Entropy Measures

Let *X* be a discrete random variable taking values in an alphabet of size *M*, with the probability mass function ${\left\{p_{i}\right\}}_{i=1}^{M}$. In the example studied, M=49 (the lottery number range). In what follows, we use small worked numerical examples from the Loto 6/49 data to make each measure tangible.

#### 2.2.1. Shannon Entropy

Shannon entropy is the average information content per alphabet symbol:


(1) $$H\left(X\right)=-\sum_{i=1}^{M}p_{i}\log_{2}p_{i}$$

For a uniform distribution, $H=\log_{2}M=H_{\max}$. In our case $H_{\max}=\log_{2}49\approx 5.6147$ bits. Shannon entropy treats symbols as exchangeable: it measures only how often each symbol appears, not the order in which they come [18].

Worked example. In the Loto 6/49 historical dataset analyzed in this study, number 1 was drawn 306 times in 15,228 observations, giving $p_{1}=0.0201$. Number 10 was drawn 316 times, giving $p_{10}=0.0208$. For a perfectly uniform lottery we would expect $p_{i}=1/49\approx 0.0204$ for every *i*. By adding the full empirical distribution into (1), we obtain H=5.6124 bits, which is 99.96% of $H_{\max}$. The small gap of 0.0023 bits comes from the minor over- and under-representation of individual numbers. If the lottery was biased/rigged, and we were stuck at drawing only number 1 (or *i* in general), we would have $p_{1}=1$ and H=0, which is the minimum value of the Shannon entropy.

#### 2.2.2. Rényi Entropy

Rényi entropy of order q≠1 [19] is a one-parameter family of entropies that generalises the Shannon entropy:


(2) $$H_{q}\left(X\right)=\frac{1}{1-q}\log_{2}\sum_{i=1}^{M}p_{i}^{q}$$

In the extreme case q→1 it gives the same result as the Shannon entropy. Different values of *q* weight the distribution in a different manner. For q<1, rare symbols count more towards the final result. For q>1, frequent symbols dominate the outcome. Analyzing several values of *q* gives a richer picture than a single Shannon number alone. This perspective leads to a spectrum representation: a source with a flat spectrum across *q* is closer to uniform than one that only happens to score well at q=1.

Worked example. One can consider the Loto 6/49 empirical distribution. Shannon entropy (q=1) is equal to 5.6124 bits, as above. Rényi at q=2 gives $H_{2}=-\log_{2}\sum_{i}p_{i}^{2}=5.6100$ bits. The gap between the two is only 0.0024 bits, so the distribution is almost as flat from the q=2 perspective as it is from the Shannon perspective. For the logistic map the same comparison gives Shannon = 5.3325 bits but $H_{2}=4.9532$ bits, a gap of 0.38 bits. The Rényi spectrum already shows that the logistic map has its probability mass more concentrated on a few symbols compared to what the Shannon entropy alone suggests.

#### 2.2.3. Permutation Entropy

Permutation entropy [2] takes into account the order in which values appear, not the values themselves. Considering the example of a sequence $\left\{x_{t}\right\}$ and an embedding dimension *m*, we slide a window of length *m* through the sequence. For each window we record the ordinal pattern—the permutation that ranks the *m* values. There are m! possible permutations. We then count how often each permutation occurs and compute the Shannon entropy of that count distribution:


(3) $$H_{\mathrm{PE}}\left(m\right)=-\sum_{\pi \in S_{m}}p\left(\pi \right)\log_{2}p\left(\pi \right)$$

The normalised version $H_{\mathrm{PE}}\left(m\right)/\log_{2}\left(m!\right)$ is equal to 1 for a fully random sequence and is a value smaller than 1 when the sequence has a specific ordinal structure. One advantage of this measure is that it is invariant under monotone transformations of the amplitudes, so it is not affected by scaling or shifting the values.

Worked example. Take m=3, so there are 3!=6 possible ordinal patterns. Consider the first five lottery numbers of the draw from 8 August 1993: 31, 42, 43, 3, 47. The first window (31, 42, 43) is strictly increasing, which is the pattern π=(1,2,3). The second window (42,43,3) has ranks (2,3,1): the largest is in the middle, the smallest at the end. The third window (43,3,47) has ranks (2,1,3). For a truly random sequence each of the six patterns should occur with probability close to 1/6, giving $H_{\mathrm{PE}}=\log_{2}6\approx 2.585$. For the logistic map at m=4 the deterministic structure shows up immediately: only a subset of the 24 possible patterns actually occurs, and $H_{\mathrm{PE}}$ drops to 3.53 out of 4.585 (about 77% of maximum).

#### 2.2.4. Sample Entropy

Sample entropy [3] asks a more demanding question: if two short segments of the sequence are close to each other within a tolerance *r*, how likely is it that they stay close when we extend both by one more symbol? Formally,

(4) $$\mathrm{SampEn}\left(m,r\right)=-\ln\frac{A}{B}$$

where *B* counts template matches of length *m* and *A* counts template matches of length m+1, both excluding self-matches. Large values mean that short patterns do not predict what comes next—the sequence is irregular. White noise typically gives SampEn≈2.2 for m=2 and r=0.2σ. Deterministic chaotic signals give lower values because patterns repeat.

Worked example. On a sample of 700 lottery numbers we obtain SampEn=2.334, slightly above the white-noise reference. On the same-length segment of the logistic map we obtain 0.666: more than three times lower. The logistic map reuses short sequence patterns much more often than the lottery does.

## 3. Dataset and Reference Sources

### 3.1. Romanian Loto 6/49

The dataset that we analyzed contains all official draws of the Romanian Loto 6/49 system (Loteria Română) from 8 August 1993 to 11 April 2026. After removing incomplete records, we are left with *N* = 2538 draws and 15,228 individual number observations. In each draw, 6 distinct numbers are selected without replacement from {1,…,49}, giving a combinatorial space of 496=13,983,816 possible outcomes. All entropy analyses in this paper treat the individual drawn numbers as symbols from the marginal alphabet {1,…,49}; the entropy of complete draw combinations would require a symbol space of size 496 and is not addressed here.

Basic uniformity checks are valid. A chi-squared test on the 49 number frequencies gives $\chi^{2}=49.30$ (p=0.421, 48 degrees of freedom). Shannon entropy is equal to 5.6124 bits, which is 99.96% of $H_{\max}=5.6147$ bits. Autocorrelation at lags up to 15 stays below 0.022 as absolute value. The numbers are consistent with those reported in [4] for a shorter cut of the same dataset (2510 draws): the 28 additional draws do not change any conclusion, which is expected.

### 3.2. Reference Sources

We build three additional sequences of the same length (15,228 values) and the same alphabet {1,…,49}:

- **PRNG.** Integers drawn uniformly at random with NumPy’s PCG64 generator [20], seeded at 42. PCG64 is a high-quality general-purpose PRNG that passes the full NIST 800-22 battery; it is not a cryptographically secure PRNG (CSPRNG) in the formal sense, but it serves as a strong statistical reference for this study.
- **Logistic map.** The recurrence $x_{t+1}=rx_{t}\left(1-x_{t}\right)$ with r=3.9999 and $x_{0}=0.1$, discretised as $\left\lfloor 1+48x_{t}\right\rfloor$. At this parameter the map is fully chaotic (positive Lyapunov exponent). Its invariant measure on [0,1] is the arcsine distribution, not uniform; however, after discretisation to M=49 bins the resulting symbol frequencies are approximately equal, which is why its Shannon entropy is close to the maximum.
- **Biased.** A fixed non-uniform distribution with relative weight 3 on numbers 1–10, weight 0.5 on 11–25, and weight 0.2 on 26–49. This is the low-entropy reference; we use it to check that the four measures respond in a predictable manner when the input is visibly non-uniform.

The logistic map is the most interesting of the three. Its Shannon entropy would pass an independence test based entirely on this value (pointing towards a pseudo-random generator), and yet it is fully deterministic. All four sources are plotted side by side in every figure.

## 4. Combined Entropy Profile

### 4.1. Main Results

Table 1 reports the four measures for all four sources. Shannon and Rényi-2 are in bits. Permutation entropy uses m=4 and is reported both in bits and as a fraction of $\log_{2}\left(4!\right)=4.585$ bits. Sample entropy uses m=2 and r=0.15σ.

The logistic map reaches 94.97% of the Shannon maximum, which on its own looks very good. Yet on permutation entropy it drops to 77.01% and on sample entropy to 0.666. The lottery, by contrast, scores 99.96%, 99.98%, and 2.334 on the same three measures. The ordinal and deterministic structure that the logistic map carries is invisible to Shannon but clearly visible to the other two.

The lottery and PRNG perform similarly on all four measures—within 0.05% in normalised terms. The biased source is the opposite of the logistic map: it has a small score based on the Shannon entropy (84.61%) but a near-maximum permutation entropy (99.70%), because the order in which its (biased) symbols occur is itself random. The four measures really do measure different things, which is why combining them is useful.

**Figure 1:**
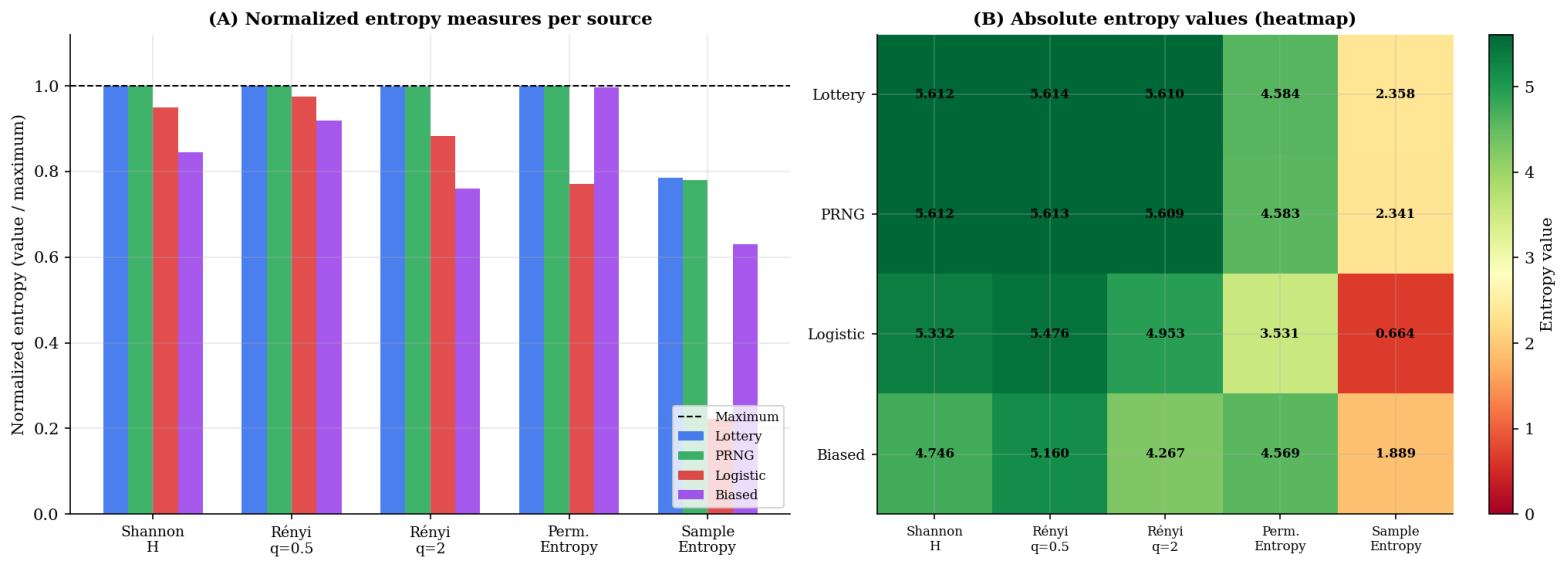
Entropy profile across all four sources.

Figure 1 shows normalised entropy values (panel A) and absolute entropy values as a heatmap (panel B) for all four sources across five measures. Lottery and PRNG trace nearly identical curves; the logistic map shows visibly lower permutation and sample entropy despite its high Shannon value.

### 4.2. Rényi Spectrum

Reading $H_{q}$ for several values of *q* gives more information than a single q=1 number. If the probability distribution is approximately uniform, $H_{q}$ is nearly constant across *q*. If the distribution is concentrated on a few symbols, $H_{q}$ drops quickly as *q* grows, because large *q* gives more weight to the high-probability symbols.

**Figure 2:**
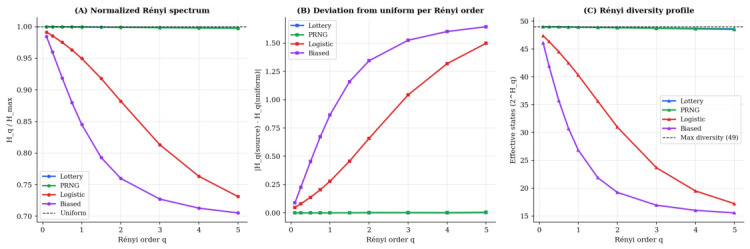
Rényi spectrum for q∈[0.1,5].

Figure 2 shows the spectrum for q∈{0.1,0.25,0.5,1,2,3,4,5}. Panel (A) plots the normalised spectrum; panel (B) the absolute deviation from the uniform reference; panel (C) the Rényi diversity profile $2^{H_{q}}$. Lottery and PRNG remain near the maximum across all *q*; the logistic map falls off past q=1 and its effective alphabet drops below 30 symbols at q=5.

## 5. Entropy Convergence and the Power-Law Check

### 5.1. Convergence Curves

We compute the Shannon entropy ratio $H\left(n\right)/H_{\max}$ as a function of the number of draws *n*, advancing in steps of 5. For the lottery and PRNG the ratio climbs quickly and monotonically toward 1: 99.85% already at n=500 draws, 99.89% at n=1000. For the logistic map the ratio stabilises at 94.97% and does not move further. For the biased source it stabilises at 84.61%.

**Figure 3:**
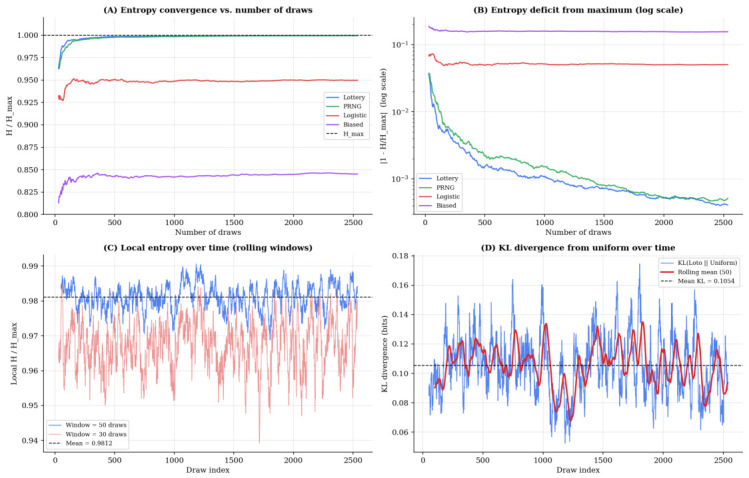
Entropy convergence and temporal dynamics.

Figure 3 has four panels. Panel (A): entropy ratio $H\left(n\right)/H_{\max}$ vs. number of draws. Panel (B): entropy deficit $|1-H\left(n\right)/H_{\max}|$ on a log scale. Panel (C): rolling entropy estimates using windows of 50 draws (blue) and 30 draws (red); the reported mean of 0.981 corresponds to the 50-draw window. Panel (D): Kullback–Leibler divergence from uniform in the same rolling windows, fluctuating around 0.1054 with no temporal trend over the 32-year period.

### 5.2. Power-Law Decay of the Entropy Deficit

Let $\delta \left(n\right)=1-H\left(n\right)/H_{\max}$ be the entropy deficit at *n* draws. We fit a power law $\delta \left(n\right)∼Cn^{\alpha }$ in log-log space on the first 150 data points, chosen to cover the early convergence regime where the deficit is large enough to fit reliably while excluding the noise floor at large *n*. The fitted exponents are:


(5) $$\alpha_{\mathrm{Loto}}=-0.958,\;\alpha_{\mathrm{PRNG}}=-0.942,\;\alpha_{\mathrm{Logistic}}=-0.066$$

**Figure 4:**
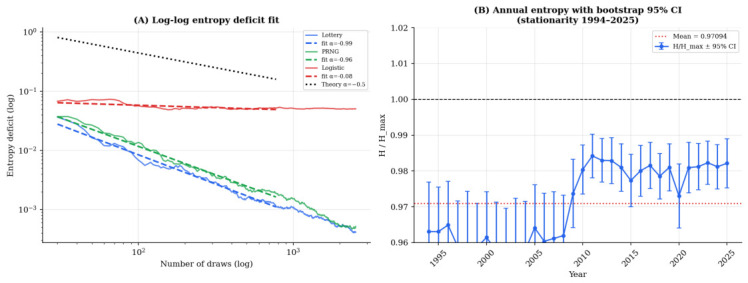
Power-law convergence and annual stationarity.

Figure 4, panel (A) shows the log-log plot of entropy deficit vs. draws; dashed lines are power-law fits and the dotted line is the $O(1/\sqrt{n})$ reference. Panel (B) shows annual Shannon entropy ratios with 95% bootstrap confidence intervals (200 resamples per year); no year is a statistical outlier.

The observed exponents α≈−0.96 for the lottery and PRNG are steeper than the classical $O(1/\sqrt{n})$ bound (α=−0.5). We note that plug-in Shannon estimators are known to be biased for finite alphabets [7], and the faster-than-$1/\sqrt{n}$ rate may partly reflect this bias rather than a genuine property of the source; the important contrast remains: the logistic map sits at |α|≈0.07, an order of magnitude below both random sources.

A practical reading: a source with α<−0.5 is shrinking its entropy deficit at a rate consistent with a genuinely random source. A structured source shows up here as a flat curve with |α| close to 0, well before its Shannon plateau is visible on a linear plot. This is a trajectory-based check rather than a single-point measurement, and in our data it separates genuine from structured sources by more than a full order of magnitude in α.

### 5.3. Local Stationarity

The rolling entropy ratio in the window of 50 draws (Figure 3, panel C) varies narrowly around a mean of 0.981, with a coefficient of variation of 0.37% and no trend over 32 years. Reducing the window to 30 draws (shown in red) increases the variance as expected from smaller samples, but the process remains stationary. Formal stationarity testing is beyond the scope of this study; the visual inspection and stable coefficient of variation are reported as descriptive evidence only.

The KL divergence from uniform in the same rolling windows (Figure 3, panel D) has a mean of 0.1054 and a standard deviation of 0.0202, again with no visible temporal structure. The annual estimates in Figure 4, panel (B), confirm this at the aggregate level: no year is an outlier.

## 6. Permutation Entropy in Detail

Permutation entropy deserves its own section because it gives the sharpest separation between the logistic map and the two genuine random sources. The lottery numbers in each draw are published in ascending order by Loteria Română; for the permutation entropy analysis we concatenate the six numbers of each draw in that published order, keeping the procedure identical for all sources. To assess sensitivity, we verified that sorting in descending order yields differences in normalised permutation entropy below 0.1% for the lottery.

**Figure 5:**
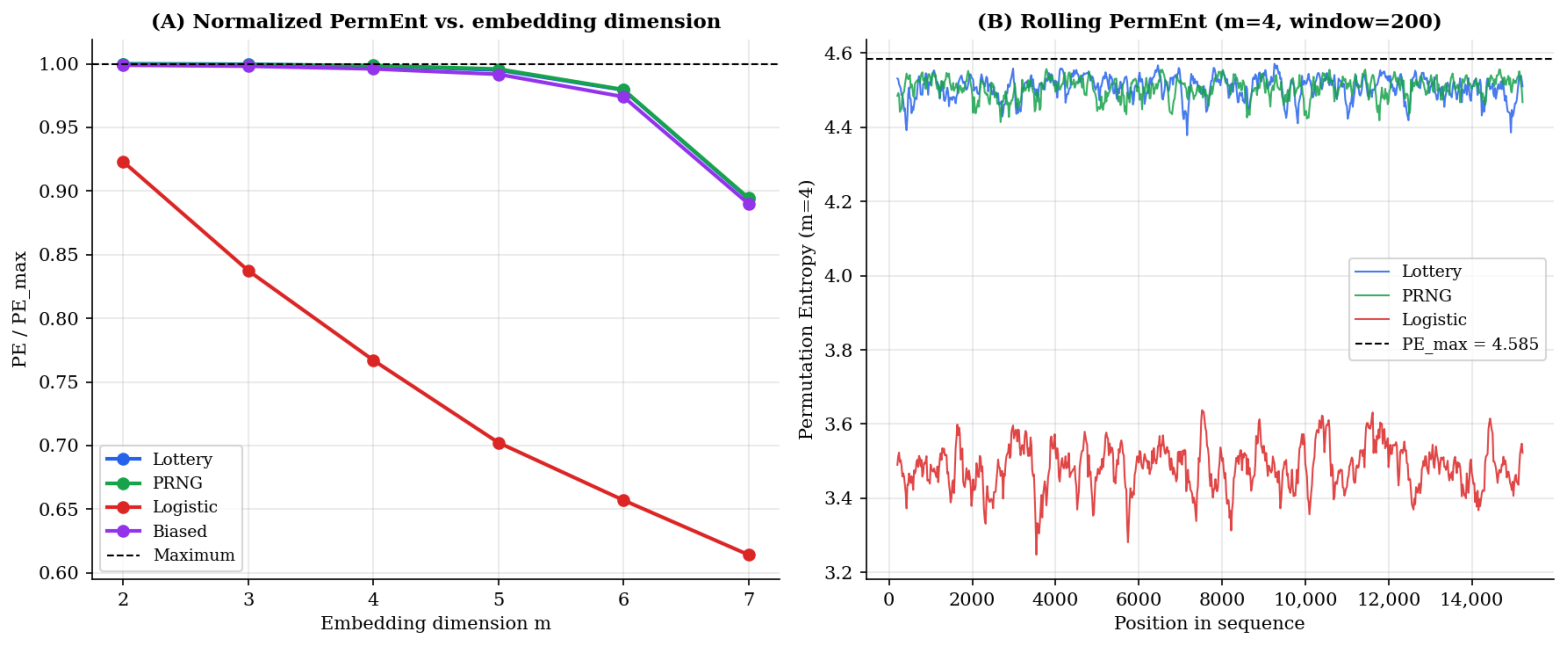
Permutation entropy across embedding dimensions and over time.

Figure 5, panel (A) plots normalised permutation entropy $H_{\mathrm{PE}}\left(m\right)/\log_{2}\left(m!\right)$ vs. embedding dimension m∈{2,…,7}. Panel (B) shows rolling permutation entropy (window of 200 numbers, m=4). Lottery and PRNG stay above 99% at all *m* and overlap in the rolling plot; the logistic map declines from m=3 and its rolling values are systematically lower and more volatile.

## 7. Machine Learning Classification

### 7.1. Setup

To check whether the entropy profile automatically separates sources, we trained a Random Forest classifier [21] using only entropy-derived features. For each source, we extracted overlapping windows of 300 consecutive symbols with a step of 30, giving approximately 488 feature vectors per source. Each feature vector is

(6) $$f=\left[H/H_{\max},\;\mathrm{KL}\left(p∥u\right),\;H_{2},\;H_{\mathrm{PE}},\;\sigma_{p},\;p_{\max},\;p_{\min},\;\left|\left\{i:p_{i}=0\right\}\right|\right]$$

where *u* is the uniform distribution, and $\sigma_{p}$ is the standard deviation of the empirical frequency vector. We used 200 trees and 5-fold stratified cross-validation, with features scaled to zero mean and unit variance.

### 7.2. Results

On the four-class task the classifier reaches 78.02 ± 2.14% accuracy, well above the 25% chance baseline. The high overall accuracy is driven primarily by the near-perfect separation of the logistic map and the biased source from the rest; the lottery–PRNG sub-task is the only difficult pair, as shown in Table 2.

On the pairwise lottery-versus-PRNG task, the accuracy is 55.74 ± 3.95%, statistically indistinguishable from the 50% baseline. Every entropy-based feature we tried gave the same answer: the two sources are equivalent under this profile.

**Figure 6:**
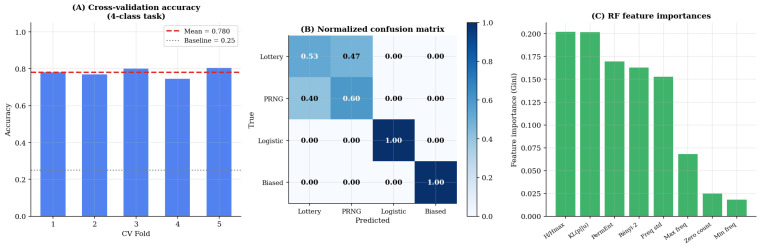
Random Forest classification from the entropy profile.

Figure 6: panel (A) cross-validation accuracy per fold (mean 78%, random baseline 25%); panel (B) normalised confusion matrix—errors occur only in the lottery–PRNG block; panel (C) feature importances—KL divergence and Rényi $H_{2}$ are the most discriminative, Shannon entropy alone the least.

## 8. Discussion

### 8.1. Shannon Is Necessary but Not Sufficient

The logistic map analysis is the main takeaway of this paper. A source that reaches 94.97% of the Shannon maximum would pass informal randomness thresholds in many applied contexts. The same source scores 77.01% on permutation entropy and 0.666 on sample entropy—two clear signals of deterministic structure.

This observation holds for the specific case studied here. More generally, a deterministic dynamical system with sufficient complexity or noise may not be detectable by finite-data entropy measures, especially if the ordinal patterns are nearly uniformly distributed or the embedding dimension is poorly chosen. The results presented are therefore illustrative of a class of structured sources rather than a general result. Shannon entropy only sees the marginal distribution of symbols. Permutation entropy and sample entropy see ordering and templates that do match. A source can have a uniform marginal distribution while being fully predictable from its history, and Shannon entropy cannot tell the difference if not corroborated with additional measures.

### 8.2. The Profile as a Practical Screen

The four measures are easy and have a low cost to compute. Shannon, Rényi, and permutation entropy run in O(n) or O(nlogn). Sample entropy is $O(n^{2})$ in the naive implementation used here, but can be approximated with *k*-d trees. On a sequence of 15,000 symbols the full profile takes less than ten seconds on a standard laptop.

For RNG validation, the profile and framework proposed in this paper can serve as a fast first pass before the full NIST 800-22 battery [5]. A source that fails on any of the four measures can be rejected immediately without running the complete suite. A source that passes all four goes on to the full battery with higher prior confidence. Passing this profile does not replace NIST SP 800-22 testing: the entropy measures evaluated here do not cover all the statistical properties tested by the full battery, and high entropy values do not imply independence in the strict statistical sense. The approach does not depend on the alphabet: we demonstrate it on an alphabet of size 49, but it applies directly to binary sequences (for hardware RNG testing), to byte sequences (for stream-cipher evaluation), and, with appropriate discretisation, to continuous-valued sources.

### 8.3. The Convergence Check

The power-law exponent *α* tells us something that a single entropy value cannot: how *fast* the source is approaching maximum entropy. A structured source reveals itself through a flat convergence curve (small |α|) long before its Shannon plateau is clearly visible.

This is useful when sample collection and processing are expensive. For a newly deployed hardware RNG or a physical generator with a slow sampling rate, waiting for Shannon entropy to stabilise near $H_{\max}$ may take a long time. Fitting a power law on early data and checking whether α<−0.5 offers a quicker conclusion.

### 8.4. Lottery Regulation

The results reinforce the conclusions of [4]. The Romanian Loto 6/49 system cannot be distinguished from a high-quality PRNG on all four entropy measures. Its convergence exponent is α=−0.958, and its KL divergence from uniform shows no trend over 32 years. Regulators can apply the same profile to other lottery and gambling systems, and the convergence check gives a way to monitor integrity over time.

### 8.5. Limitations

The method applies directly to any discrete source with a finite alphabet. Extending it to continuous sources requires a discretisation step, and the choice of bin width affects absolute entropy values. The relative comparisons between different sources are robust to this choice as long as the same bins are used for all sources.

Sample entropy scales as $O(n^{2})$ in the implementation used here, so we limited that computation to subsequences of length 700. Approximate estimators based on *k*-d trees or GPU acceleration reduce this to O(nlogn) and would allow full-sequence analysis.

The logistic map at r=3.9999 is close to the fully chaotic regime and its invariant measure is nearly uniform, which makes it the hardest case for Shannon entropy. Other chaotic maps (tent map, Hénon map) have stronger non-uniformities and would be easier to detect. The biased reference is a simple static non-uniform distribution; Markov-generated or hidden Markov sequences would give profiles somewhere in between, and would be a natural extension of this study.

## 9. Conclusions

Shannon entropy alone is not a sufficient measure to state with absolute certainty that a source is random. A logistic map at r=3.9999 reaches 94.97% of the Shannon maximum but only 77.01% on permutation entropy and 0.67 on sample entropy. The Romanian Loto 6/49 lottery reaches 99.96%, 99.98%, and 2.33 on the same measures.

Combining Shannon, Rényi, permutation, and sample entropy into a single profile puts the lottery profile very close to a high-quality PRNG. A Random Forest classifier trained on the entropy profile is capable of separating the logistic map and a biased source from the rest with near-perfect accuracy, but cannot distinguish between lottery and PRNG at the chance level, confirming that the two sources are equivalent under this battery of tests.

The entropy deficit of both the lottery and the PRNG decays as a power law with exponent α≈−0.96, faster than the classical $O(1/\sqrt{n})$ bound. The logistic map sits at α≈−0.07. The gap is large and the check is not expensive, so fitting α on early data gives a simple way to flag structured sources.

The approach is alphabet-independent and directly applicable to PRNG validation, hardware RNG certification, cryptographic key generation audits, and detection of structured pseudo-random generators in any discrete setting.

## Figures and Tables

**Table 1:** Entropy profile across four sources.

Source	Shannon *H* (bits)	Rényi *H*_2_ (bits)	PermEnt (*m* = 4)	SampEn
Lottery (Loto 6/49)	5.6124 (99.96%)	5.6100	4.5839 (99.98%)	2.334
PRNG (PCG64)	5.6118 (99.95%)	5.6089	4.5828 (99.95%)	2.291
Logistic (*r* = 3.9999)	5.3325 (94.97%)	4.9532	3.5310 (77.01%)	0.666
Biased	4.7463 (84.61%)	4.2673	4.5692 (99.70%)	1.886
Maximum	5.6147	5.6147	4.5850	—

Values in parentheses are percentages of the maximum. Sample entropy reference: white noise gives ≈2.2.

**Table 2:** Normalised confusion matrix (rows: true class, columns: predicted class).

	Lottery	PRNG	Logistic	Biased
Lottery	0.53	0.47	0.00	0.00
PRNG	0.40	0.60	0.00	0.00
Logistic	0.00	0.00	1.00	0.00
Biased	0.00	0.00	0.00	1.00

Logistic map and biased source are classified almost perfectly. Lottery and PRNG are confused with each other about as often as the classifier guesses.

## Data Availability

The Loto 6/49 historical data is publicly available from Loteria Română (https://www.loto.ro and was accessed on 10 May 2026). All Python analysis code can be made available to any interested researcher.
